# Transformation of human embryonic stem cells by HTLV-1 Tax

**DOI:** 10.1186/1742-4690-8-S1-A164

**Published:** 2011-06-06

**Authors:** Linda Zane, Junichiro Yasunaga, Takao Kinjo, Gustavo Brito de Melo, Kuan-Teh Jeang

**Affiliations:** 1 National Institute of Allergy and Infectious Diseases, NIH, USA

## 

HTLV-1 encodes a viral oncoprotein Tax which has been shown to transform primary rodent cells. Tax can also immortalize human primary T lymphocytes. However, to date transformation by Tax of human cells has been unsuccessful [1,2]. Based on the cancer stem cells (CSCs) hypothesis that tumor growth is caused by the proliferation of only a small number of CSCs, we reasoned that ATL cells may arise from the infection by HTLV-1 of not mature CD4+ T cells (as currently thought) but by viral infection of more rare hematopoietic precursor cells. Thus, Tax may be able to transform early undifferentiated human cells, but not later differentiated cells. We asked whether Tax could transform human Embryonic Stem Cells (ESCs) vs. human skin fibroblast BJ cells. Undifferentiated ESCs were transfected with Tax, and these cells were injected into immunodeficient NOD-SCID/IL2γR null (NOG) mice. We assessed tumor incidence after injection and found that Tax enhanced the tumorigenesis of ESCs (p=0.012, Gehan-Breslow-Wilcoxon test). By contrast, our transformation studies using Tax in differentiated human BJ cells have been consistently unsuccessful. Based on these results and others to be presented, we conclude that HTLV-1 Tax is more capable of inducing tumorigenesis of undifferentiated than differentiated human cells.

## References

[B1] Higuchi M Fujii M Distinct functions of HTLV-1 Tax1 from HTLV-2 Tax2 contribute to key roles in viral pathogenesis Retrovirology 2009 6 117 10.1186/1742-4690-6-117 20017952 PMC2806368

[B2] Matsuoka M Jeang KT Human T-cell leukemia virus type 1 (HTLV-1) and leukemic transformation: viral infectivity, Tax, HBZ and therapy Oncogene 2011 Epub a head of print 21119600 10.1038/onc.2010.537 PMC3413891

